# Toxicity bioassay and sub-lethal effects of profenofos-based insecticide on behavior, biochemical, hematological, and histopathological responses in Grass carp (*Ctenopharyngodon idella*)

**DOI:** 10.1007/s10646-023-02628-9

**Published:** 2023-01-28

**Authors:** Zeinab. M. El-bouhy, Fatma. A. S. Mohamed, Mohamed. W. A. Elashhab, Walaa El-Houseiny

**Affiliations:** 1 Aquatic Animal Medicine Department, Faculty of Veterinary Medicine, Zagazig University, Zagazig, 44511 Egypt; 2 National Institute of Oceanography and Fisheries (NIOF), Cairo, Egypt

**Keywords:** Profenofos, LC50, Leukocytosis, Grass carp, Tissues alterations

## Abstract

Profenofos (organophosphate) is among the major toxicant polluting freshwater bodies, exerting a significant effect on fish health. The LC_50_ value of Profenofos (PRO) was resolved in Grass carp (*Ctenopharyngodon idella*) with average body weight (55.82 ± 5.42 g) and determined the 96 h LC_50_ value as 7.2 µg/L for the assay. Twenty-one-day exposures to 1.8 µg/ L and 3.6 µg/ L doses were conducted to evaluate the sub-lethal effects, and various toxicological endpoints were assessed on the 1^st^, 7^th^, 15^th^ and 21^st^ days of exposure. Acute toxic stress was observed with fish displaying behavioral toxicity. The most hematological change was extreme microcytic hypochromic anemia. Leucocyte count increased in experimented fish. Moderate neutrophilia, monocytosis and lymphocytosis were observed. Serum total protein, albumin, and globulin concentrations were significantly diminished. Overall, increments over control were recognized in serum urea, creatinine and acid phosphatase. However, serum glucose, total lipid, cholesterol, serum ALT and AST activity showed a significant decrease in fish exposed to both concentrations of PRO. Serum IgM concentrations insignificantly changed in treated fish except for on the 21^st^ day of exposure to 3.6 µg/ L of PRO, while serum lysozyme significantly decreased. Furthermore, total protein, lipid and glycogen concentrations in muscles and the liver exhibited a decreasing trend at all concentrations. Moreover, histopathological alterations in the liver, kidney, and muscles occurred exclusively after treatment. From the obtained results, it is assumed that profenofos induced general toxic impacts under field conditions and might disturb ecologically relevant processes.

## Introduction

Industrialization and technological advancement caused the introduction of chemicals such as agrochemicals, pesticides, halogenated polycyclic hydrocarbons and food additives (Ibeto and Okoye 2010). The environmental consequences of these substances have become a worldwide concern. Pesticides are one of the most efficient weapons that man has devised to protect agricultural products from pest attacks (El-Houseiny et al. 2022a). Although there is a lot of research in the field of pesticides, the quantity of knowledge accessible about the effects of specific pesticides on non-target organisms varies greatly.

Pesticides constitute a persistent hazard to aquatic life by affecting habitat, behavior, growth, and reproductive potential (Ibrahim et al. 2019; Taha 2022). Fish are extremely sensitive to changes in their habitats and can help to determine the risks associated with new chemical contamination in aquatic ecosystems (El-Houseiny et al. 2022a; Khalil et al. 2022). They provide a significant portion of global human nutrition, so protecting fish health is critical.

Profenofos (PRO) is an organophosphate insecticide that is utilized in agriculture to control insects. It has a short persistence and is easily decomposed; hence it was extensively applied in Egypt during those years for selective mite control on cotton, maize, and other vegetables (Sharafeldin et al. 2015). The solubility of profenofos was confirmed by HPLC, dissolved in the solvent, and tested water samples at a concentration of 1 ppm (Ghazla et al. 2019). PRO, like other insecticides, may find its way into water systems, causing harm to aquatic life. It causes substantial harm to aquatic species and even endangers human safety throughout the food chain. PRO induced marked hepatotoxic and cytotoxic impacts in *Cyprinus Carpio* (Joseph and Raj 2010). It induced alterations in the locomotor behavior and tissue architecture of the fish *Gambusia affinis* (Rao et al. 2006). Moreover, it had adverse biochemical and histopathological impacts on the liver and kidneys of white egrets, *Egretta alba* (Taha 2022).

Toxicity testing is critical for determining the impact and fate of toxicants in aquatic habitats. The acute toxicity trials on zebrafish (*Brachydanio rerio*), tilapia (*Oreochromis mossambicus*), and common carp (*Cyprinus carpio L*) have been estimated earlier (Min and Cha 2000; Rao et al. 2003; Ismail et al. 2009). It is extremely hazardous to crustaceans (Blue crab, *Callinectes sapidus*, has an LC_50_ value of 33.0 µg/L), zooplankton (Immature scud, *Gammarus pseudolimnaeus*, has a 96 h LC_50_ value of 1.30 µg/L), Bluegill (300 µg/L), Crucian carp (90.0 µg/L), and Rainbow trout (80.0 µg/L) (Tomlin 1994). 62.4 µg/L was a 96 h LC_50_ value for common carp (Ismail et al. 2009), and 0.057 mg/L was LC_50_ for zebrafish (Sultana et al. 2021). However, studies on PRO-mediated toxicities in grass carp are still scanty. Grass carp (*C. idella*) is a freshwater fish that has a place with the family *Cyprinidae*. It has a fast growth rate and high commercial values (Wang et al. 2008 and Qu et al. 2016). They effectively control aquatic vegetation plants (Cudmore et al. 2017). The impacts of contaminants on fish survival rates and health, as well as human health, have generated cause for concern due to the proliferation of contamination in waters.

Therefore, given the ecological impact of this pesticide, this study was carried out to explore the effect of PRO on the health status of grass carp (*C. idella*). For achieving the previous objective, the LC_50_ of PRO after 96 h was determined. Moreover, the sub-lethal effect of 25 and 50 % of PRO LC_50_ was assessed on hematological, biochemical, and immunological parameters as well as histopathological findings in certain organs in grass carp (*C. idella*) after 1, 7, 15 and 21 days of exposure.

## Material and method

### Test compounds, reagents, and chemicals

Profenofos (Ictacrune) (Nagarjuna, India) is a broad-spectrum organophosphate insecticide widely used to control *Lepidoptera* in cotton and soybean with strong effects against mining and sucking insects as well as mites. Each liter of Ictacrune contains 72 g of the active substance (profenofos). The total amount of PRO to be added was calculated after each aquarium’s volume was determined. All other reagents, chemicals, and stains used were purchased from (Sigma, St.Louis, MO) and were of analytical grade

### Experimental fish

A total of 160 apparently healthy live grass carp *(C. idella*) with an average body (55.82 ± 5.42 g) were obtained from the private fish farm at Damietta province, Egypt. Fish were obtained to determine 96 h LC_50_ and the sublethal toxicity effect of PRO. Fish were transported to the laboratory of the National Institute of Oceanography and Fishers, Al-Qanater branch, Egypt. The fish were acclimated to the laboratory conditions for two weeks. They were fed commercial pellets daily at 3% body weight (BW) during acclimatization two times daily at 9:00 and 16:00 h.

### Experimental studies

#### Determination of 96 h LC_50_ of PRO (acute toxicity test)

The test individuals were exposed to selected and serially diluted PRO concentrations as shown in Table 1. Acute toxicity bioassay was conducted with a definitive test in a semi-static system in the laboratory as per the standard methods (APHA 2005). A total of 70 grass carp *(C. idella*) were divided into 7 groups (10 fish/group). The first group was left as a control while 2^nd^, 3^rd^, 4^th^, 5^th^, 6^th^, and 7^th^ were exposed to 2, 4, 6, 8, 10 and 12 µg/l of PRO, respectively. These concentrations were selected to determine 96 h LC_50_ of PRO. Fish were observed at 12 h intervals up to 96 h. The water parameters were within the recommended ranges during the experiment (pH = 7.2 ± 0.5; ammonia = 0.02 ± 0.001 mg/L, nitrite = 0.017 ± 0.001 mg/L; dissolved oxygen 6.55 mg/L; water temperature 24 °C; photoperiod 12:12 light: dark). No feed was given during the test period. Dead fish were removed immediately upon discovery. Mortalities and survival times were recorded, and then LC_50_ was calculated according to the equation (Behrens and Karper 1953) (Table 2).

**Table 1:** Preliminary trials for zero and a hundred % mortalities in grass carp *(C. idella)* exposed to different concentrations of PRO (After 96 h)

Group number (N = 10)	Concentrations of profenofos µg/l	Mortality number during 96 h				Total mortality number	Total mortality %
		1^st^ day	2^nd^ day	3^rd^ day	4^th^ day		
1	Control	0	0	0	0	0	0
2	2	0	0	0	0	0	0
3	4	0	0	0	1	1	10
4	6	0	0	1	2	3	30
5	8	0	1	2	3	6	60
6	10	0	2	3	4	9	90
7	12	0	3	4	3	10	100

**Table 2:** Actual estimation of 96 h mortalities LC_50_ in grass carp *(C. idella)* exposed to different concentrations of PRO

Groups (N = 10)	Concentrations µg/l	No. of dead fish at 96 h	a	b	axb	∑ axb
2	2	0				
3	4	1	2	0.5	1	
4	6	3	2	2	4	
5	8	6	2	4.5	9	
6	10	9	2	7.5	15	
7	12	10	2	9.5	19	
						48

96 h LC_50_ = Highest dose - ∑ axb/n

a = constant factor between two successive doses

b = the mean of dead fish in each group

∑ axb = sum of axb

n = number of fish in each group

= 12 – 48/10

= 12 – 4.8

= 7.2 µg/l

#### Effect of Sub-lethal toxicity of PRO in *C. idella*

For sub-lethal toxicity tests, the fish were grouped into three batches. Each batch had 10 fish and had 3 replicates. PRO was prepared to produce the required concentration (25% (1.8 µg/ L) and 50% (3.6 µg/ L) of 96 h LC_50_) in which 96 h LC_50_ of PRO = 7.2 µg/ L. The media was renewed every alternate day. Fish were fed daily with 3% of body weight. The amount of feed was readjusted every week according to the biomass of each replicate throughout the experiment; the uneaten feed was collected by siphoning. The proximate analysis of the basal diet indicated 38.9% crude protein, 10.5% crude lipid, and 3.68% fiber, according to NRC (2011). After their respective exposure, the fish were observed daily for 21 days for any alterations in behavior. The mortalities, clinical signs, and postmortem findings were recorded.

### Sampling

On the 1^st^, 7^th^, 15^th^ and 21^st^ days of exposure to PRO, blood samples were collected from the caudal veins of (5 fish at each time from each group, 0.5–0.0.8 ml from each fish) and were divided into two parts. The first part was collected into plain, clean, and sterile centrifuge tubes without anticoagulant to separate serum for biochemical analysis. The second part was taken in EDTA tubes for a complete blood cell count picture. Moreover, liver and muscle tissues were collected on the 1^st^, 7^th^, 15^th^ and 21^st^ days from exposure to PRO and stored in the freezer (at −20 °C) till used. After that, Liver and muscle samples (about 100 mg.) were homogenized, and the total proteins were precipitated by saline 0.9%, while total lipids and glycogen were precipitated by ethanol 95%. Then, centrifugation at 3000 rpm for 15 min occurred, and the supernatants were used to determine tissue biochemical parameters. Parts of the liver, Kidney, and muscles were fixed for 48 h in 10% neutral buffered formalin for histopathological examinations.

### Blood cells count picture

Red blood cell counts, hemoglobin (Hb) concentration, packed cell volume (PCV), mean corpuscular volume (MCV), mean cell hemoglobin concentration (MCHC), and white blood cell counts were assessed by using an automated blood cell analyzer of Sysmex XT-2000iVKobe (Japan) (Harvey 2012). Giemsa-stained blood smears were prepared for the differential leukocytic count, including lymphocytes, neutrophils, eosinophils, and monocytes (Dacie and Lewis 1984).

### Determination of some serum biochemical parameters

#### Liver and kidney function tests as well as immunological response

Liver and kidney injury byproducts such as alanine (ALT) and aspartate (AST) aminotransferases were determined colorimetrically using readily made kits according to the method described by Reitman and Frankel (1957). Both acid and alkaline phosphatase activities were measured using methods described by Kaplan et al. (1988) and Kind and King (1954), respectively. The serum total proteins and albumin were measured colorimetrically according to the method described by Gornall et al. (1949) and Doumas et al. (1971), respectively. Serum globulin concentration was calculated by subtracting serum albumin from total protein serum (Coles 1974). Serum creatinine was determined according to Tietz (1986). While the concentration of serum uric acid and urea were measured enzymatically according to Tietz (1990). The serum glucose, cholesterol, and total lipids were estimated according to Trinder (1969), Ellefson and Caraway (1976) and Frings and Dunn (1970), respectively. Selective immunological parameters such as IgM were measured using fish-specific ELISA kits according to the manufacturer’s instructions. Meanwhile, lysozyme activity in the serum was quantified by inhibition zone assays in agarose gel plates, as described by Mohrig and Messner (1968).

#### Determination of some tissue biochemical parameters

Total proteins, total lipids, and glycogen were estimated according to Gornall et al. (1949), Frings and Dunn (1970) and Seifter et al. (1950), respectively.

#### Histopathological studies

Samples from the liver, kidney, and muscles were collected on the 1^st^, 7^th^, 15^th^ and 21^st^ days from exposure to PRO for histopathological examinations. The collected samples were fixed in 10% buffered neutral formalin for 24 h, washed with running water, dehydrated in alcohol, and cleared in xylene and sections of 4–6 µ were prepared according to Bancroft et al. (1996).

### Statistical analysis

All data were analyzed using the statistical package for social science (SPSS 15.0 software, 2008). The total variation was analyzed by one-way variance analysis (ANOVA). Duncan’s test was used to determine significance. Probability levels of less than 0.05 were considered significant, according to Snedecor and Cochran (1987).

## Results

### Toxicity test

#### Acute toxicity (Determination of PRO lethal concentrations)

The 96-h LC_50_ of PRO for *C. idella* was calculated. Tables 1 and 2 demonstrated the mortality of *C. idella* at different concentrations of PRO. The 96-h LC_50_ was calculated, and the results showed that the 96-h LC_50_ of PRO was 7.2 µg/L.

#### Clinical signs and postmortem

*Ctenopharyngodon idella* exposed to PRO swam erratically and rapidly with semi-circular swimming behavior, trying to jump out of the aquarium, increased opercular movement, rapid gulping of water as well as knocking the wall of the aquarium, then the fish showed vertical position with head downwards, loss of equilibrium and sinking to the bottom. In later stages, the exposed excited fish laid on their sides on the bottom of the aquarium, making very slight movement and remaining motionless on the aquarium bottom until death. Exposed fish to PRO showed loss of scales, excessive mucous secretion, shining skin color, fin rot, pale gills, inflamed swim bladder and congestion of internal organs.

Fish exposed to 1.8 µg/L (25% of LC_50_) and 3.6 µg/L (50% of LC_50_) of PRO showed fast movements of the operculum during the first 7 days. Later, the operculum moved normally. At the end of the exposure period, lethargy was noticed. No mortality occurred in the aquarium. Fish exposed to PRO showed abnormal swimming on its side and sank to the bottom of the aquarium. At postmortem, fish showed excessive mucous secretion, loss of scales and shined skin color, pale gills, inflamed swim bladder, gall bladder and inflamed kidney and liver.

#### Hematological studies

The hematological parameters were tested for the surviving fish under various doses of PRO. Fish exposed to 1.8 µg/L and 3.6 µg/L of PRO showed a significant decrease in the Hb and the hematocrit value after 1,7, 15 and 21 days of exposure, reaching a minimum value on day 1 after exposure to 3.6 µg/L of PRO, compared with control (Table 3). On 1^st^ day of exposure to 1.8 µg/L of PRO, the red blood cell counts of fish showed a non-significant decrease compared to the control followed by a significant increase after exposure to 3.6 µg/L for 1^st^ day. While, on 7^th^ and 15^th^ days of exposure to both concentrations of PRO, the red blood cells count of fish showed non-significant changes compared with control (Table 3). Later, after exposure of fish to two concentration of PRO for 21 days, a significant decrease was recorded in RBCs count. MCH and MCV showed a highly significant decrease on 1^st^, 7^th^, 15^th^, and 21^st^ days, reaching to a minimum value on1^st^ day after exposure to 3.6 µg/L of PRO. MCHC were significantly decreased, reaching a minimum value after exposure to 3.6 µg/L; however, it showed a transient non-significant decrease on 7^th^ and 15^th^ day (Table 3). While on the 21^st^ day, there was a significant decrease in MCHC after exposure to 1.8 µg/L of PRO. Blood smears from different groups of fish exposed to sub-lethal concentrations of PRO revealed aberrant erythrocytic morphologies, including nuclear degeneration, micronuclear formation, binuclear development, tear drop appearance, and hypochromic erythrocytes.

**Table 3:** Effect of different concentrations of Profenofos (PRO) on hematological indices of *Ctenopharyngodon idella*

Exposure time (day)	PRO concentration	Hb (g/dl)	Ht (%)	RBCs (x10^6^/ mm^3^)	MCH(pg)	MCV (fl)	MCHC (%)	WBCs (X 10^3^ / mm^3^)	Neutrophils (X 10^3^ / mm^3^)	Lymphocytes (X 10^3^ / mm^3^)	Monocytes (X 10^3^ / mm^3^)	Eosinophils (X 10^3^ / mm^3^)
1	None (control)	5.82 ± 0.18^a^	31.37 ± 0.62^a^	1.26 ± 0.02^b^	48.70 ± 2.51^a^	241.40 ± 8.90^a^	18.60 ± 0.35^a^	13.98 ± 0.35^c^	3.60 ± 0.05^b^	9.53 ± 0.37^c^	0.33 ± 0.04^c^	0.21 ± 0.04^a^
	1.8 µg/l	2.13 ± 0.79^b^	19.40 ± 2.38^b^	1.25 ± 0.03^b^	17.00 ± 6.36^b^	154.83 ± 18.98^b^	10.36 ± 2.60^b^	39.33 ± .88^b^	11.16 ± 0.60^a^	26.72 ± 0.09^b^	1.19 ± 0.25^a^	0.23 ± 0.13^a^
	3.6 µg/l	1.50 ± 0.75 ^c^	16.80 ± 2.60^c^	1.48 ± 0.01^a^	10.03 ± 5.04^b^	112.97 ± 17.41^b^	8.06 ± 2.77^b^	51.00 ± 1.15^a^	11.93 ± 1.30^a^	37.84 ± 0.64^a^	1.03 ± 0.32^b^	0.00 ± 0.00^b^
7	None (control)	6.02 ± 0.21^a^	31.33 ± 0.60^a^	1.24 ± 0.01^a^	47.66 ± 2.42^a^	249.38 ± 9.44^a^	19.12 ± 0.34^a^	14.46 ± 0.37^c^	3.61 ± 0.05^b^	9.78 ± 0.38^c^	0.29 ± 0.03^b^	0.22 ± 0.04^a^
	1.8 µg/l	3.50 ± 1.19^b^	22.50 ± 3.57^b^	1.25 ± 0.04^a^	26.38 ± 8.47^b^	171.63 ± 22.43^b^	14.49 ± 3.26^b^	48.20 ± 0.61^b^	11.58 ± 0.87^a^	35.49 ± 0.15^b^	0.79 ± 0.31^a^	0.25 ± 0.15^a^
	3.6 µg/l	3.83 ± 0.38^b^	23.83 ± 0.84^b^	1.26 ± 0.04^a^	28.83 ± 2.07^b^	179.77 ± 0.95^b^	16.03 ± 1.09^a,b^	53.46 ± 2.07^a^	12.47 ± 0.60^a^	40.27 ± 1.57^a^	0.71 ± 0.18^a^	0.00 ± 0.00^b^
15	None (control)	6.24 ± 0.23^a^	31.39 ± 0.63^a^	1.22 ± 0.05^a^	48.48 ± 2.31^a^	254.50 ± 9.65^a^	19.40 ± 0.36^a^	13.04 ± 0.32^c^	3.56 ± 0.05^c^	9.36 ± 0.36^c^	0.30 ± 0.04^b^	0.22 ± 0.04^a^
	1.8 µg/l	4.30 ± 0.72^b^	27.73 ± 4.13^b^	1.21 ± 0.11^a^	44.96 ± 7.76^a,b^	242.13 ± 13.64^a^	18.26 ± 2.24^a^	28.00 ± 0.57^b^	5.87 ± 0.19^b^	21.18 ± 0.35^b^	0.37 ± 0.09^b^	0. 21 ± 0.09^a^
	3.6 µg/l	4.23 ± 0.41^b^	25.30 ± 1.17^b^	1.21 ± 0.04^a^	36.76 ± 2.16^b^	220.60 ± 4.22^b^	16.66 ± 0.89^b^	40.66 ± 1.20^a^	7.87 ± 0.36^a^	32.25 ± 0.92^a^	0.72 ± 0.17^a^	0.00 ± 0.00^b^
21	None (control)	5.96 ± 0.19^a^	31.28 ± 0.55^a^	1.27 ± 0.02^a^	47.30 ± 2.38^a^	246.39 ± 9.52^a^	18.46 ± 0.31^a^	14.856 ± 0.43^c^	3.62 ± 0.05^c^	9.86 ± 0.39^c^	0.31 ± 0.04^b^	0.20 ± 0.04^a^
	1.8 µg/l	2.96 ± 0.90^b^	21.20 ± 2.97^b^	0.97 ± 0.08^c^	30.53 ± 8.72^b^	219.27 ± 29.94^b^	13.30 ± 2.60^b^	69.63 ± 1.03^a^	14.64 ± 0.91^a^	54.05 ± 0.18^a^	0.69 ± 0.01^a^	0.23 ± 0.23^a^
	3.6 µg/l	3.97 ± 0.42^b^	24.23 ± 1.14^b^	1.08 ± 0.05^b,c^	36.40 ± 1.95^b^	223.77 ± 2.58^b^	16.30 ± 0.96^a,b^	48.46 ± 0.44^b^	10.08 ± 0.46^b^	37.58 ± 0.51^b^	0.16 ± 0.16^c^	0.00 ± 0.00^b^

Values are represented as the mean ± SE. The means within the same column carrying different superscripts are significantly different

*Hb* hemoglobin content, *Ht* hematocrit value, *RBCs* red blood cells, MCH mean cell hemoglobin, *MCV* mean cell volume, *MCHC* mean cell hemoglobin concentrations, *WBCs* white blood cells

Concerning white blood cell counts (WBCs), Table 3 revealed a significant increase in WBCs of *C. idella* on days 1, 7, 15 and 21 after exposure to 1.8 and 3.6 µg/L of PRO; also, there were significant increases in the neutrophils, monocytes, and lymphocytes counts of *C. idella* all over exposure periods. On the 1^st^, 7^th^, 15^th^, and 21^st^ days, eosinophil counts of *C. idella* showed non-significant changes after exposure to 1.8 µg/L of PRO. In contrast, there was a significant decrease in eosinophils counts after exposure to 3.6 µg/L all over the periods.

### Serum biochemical parameters

#### Liver and kidney function tests

This section deals with the study of the effects of sub-lethal exposure to 1.8 and 3.6 µg/L of PRO. The serum ALT and AST activity showed significant decreases in *C. idella* after exposure to both concentrations over the periods (Table 4). Significant increases were observed in the serum acid phosphatase activity of *C. idella* on days 1, 7, 15 and 21 after exposure to 1.8 and 3.6 µg/L of PRO. While on 1^st^ day, the serum alkaline phosphatase activity showed a significant increase in *C. idella*, a sudden significant decrease in its level was recorded on day 15 after exposure to 3.6 µg/L of PRO.

**Table 4:** Serum biochemical parameters of *Ctenopharyngodon idella* exposed to different concentrations of profenofos

Exposure time (day)	PRO concentration	ALT (IU/ml)	AST (IU/ml)	Alkaline phosphatase (IU/L)	Acid phosphatase (IU/L)	Total protein (g/dl)	Albumin (g/dl)	Globulin (g/dl)	Glucose (mg/dl)	Total lipid (mg/dl)	cholesterol (mg/dl)	Urea (mg/dl)	Creatinine (mg/dl)	Uric acid(mg/dl)
1	(control)	19.03 ± 2.04^b^	115.00 ± 2.32^a^	60.66 ± 4.09^b^	0.02 ± 0.002^b^	3.42 ± 0.21^a^	1.08 ± 0.20^a^	2.34 ± 0.20^a^	95.14 ± 2.04^a^	566.00 ± 33.32^a^	91.54 ± 2.01^b^	6.82 ± 0.50^b^	0.18 ± 0.02^b^	3.56 ± 0.66^a^
	1.8 µg/l	29.66 ± 7.79^a^	81.33 ± 16.89^b^	184.00 ± 37.81^a^	0.04 ± 0.003^a^	3.41 ± 0.30^a^	0.90 ± 0.09^b^	2.51 ± 0.21^a^	92.00 ± 3.05^a^	349.00 ± 18.83^b^	106.00 ± 19.50^a,b^	12.21 ± 1.43^a^	0.32 ± 0.02^a^	2.50 ± 0.28^a,b^
	3.6 µg/l	24.66 ± 7.05^a,b^	99.66 ± 4.33^b^	172.00 ± 19.34^a^	0.05 ± 0.00^a^	3.75 ± 0.14^a^	1.05 ± 0.09^a^	2.69 ± 0.10^a^	87.00 ± 2.08^b^	458.00 ± 55.11^a,b^	113.00 ± 2.60^a^	14.06 ± 1.19^a^	0.29 ± 0.03^a^	1.66 ± 0.16^b^
7	(control)	19.48 ± 2.08^a,b^	113.02 ± 2.35^a^	59.62 ± 4.07^a^	0.02 ± 0.001^c^	3.43 ± 0.21^a^	1.05 ± 0.20^a^	2.38 ± 0.20^a^	95.36 ± 2.01^a^	564.00 ± 33.34^a^	91.62 ± 2.02^a,b^	6.91 ± 0.51^b^	0.19 ± 0.02^c^	3.62 ± 0.67^a^
	1.8 µg/l	21.66 ± 1.66^a^	95.66 ± 7.83^a,b^	48.66 ± 7.79^a^	0.03 ± 0.00^b^	2.50 ± 0.14^b^	0.69 ± 0.08^a,b^	1.80 ± 0.11^b^	80.00 ± 2.00^b^	302.00 ± 71.21^b^	103.00 ± 8.00^a^	16.63 ± 1.96^a^	0.30 ± 0.02^b^	1.43 ± 0.033^b^
	3.6 µg/l	13.66 ± 1.45^b^	78.00 ± 12.12^b^	49.66 ± 6.69^a^	0.04 ± 0.001^a^	1.66 ± 0.08^c^	0.48 ± 0.09^b^	1.18 ± 0.07^c^	69.33 ± 2.03^c^	187.00 ± 36.08^b^	69.33 ± 10.11^b^	15.10 ± 0.89^a^	0.41 ± 0.002^a^	1.33 ± 0.12^b^
15	(control)	19.32 ± 2.00^a^	112.03 ± 2.36^a^	60.26 ± 4.08^a^	0.02 ± 0.002^b^	3.40 ± 0.20^a^	1.06 ± 0.20^a^	2.34 ± 0.20^a^	95.64 ± 2.02^a^	564.00 ± 33.34^a^	91.64 ± 2.01^a^	6.93 ± 0.52^b^	0.18 ± 0.02^b^	3.64 ± 0.65^a^
	1.8 µg/l	13.66 ± 2.84^b^	33.33 ± 4.09^b^	44.00 ± 7.21^a,b^	0.86 ± 0.08^a^	1.58 ± 0.13^b^	0.42 ± 0.05^b^	1.15 ± 0.10^b^	35.00 ± 1.15^b^	125.00 ± 35.94^b^	79.66 ± 5.54^b^	15.10 ± 1.37^a^	0.46 ± 0.04^a^	0.57 ± 0.09^b^
	3.6 µg/l	12.33 ± 0.66^b^	23.00 ± 2.64^b^	30.66 ± 1.20^b^	1.20 ± 0.15^a^	1.82 ± 0.09^b^	0.62 ± 0.012^b^	1.19 ± 0.09^b^	33.66 ± 4.25^b^	160.00 ± 18.65^b^	76.33 ± 4.48^b^	15.60 ± 2.38^a^	0.51 ± 0.03^a^	0.21 ± 0.04^b^
21	(control)	19.08 ± 2.02^a^	114.06 ± 2.23^a^	60.18 ± 4.08^a^	0.02 ± 0.001^b^	3.42 ± 0.20^a^	1.06 ± 0.20^a^	2.36 ± 0.20^a^	95.62 ± 2.01^a^	560.00 ± 33.38^a^	91.66 ± 2.02^a^	6.96 ± 0.51^b^	0.19 ± 0.02^b^	3.61 ± 0.66^a^
	1.8 µg/l	11.33 ± 0.88^b^	35.33 ± 4.40^b^	28.33 ± 3.17^b^	1.40 ± 0.11^a^	2.30 ± 0.57^b^	0.48 ± 0.12^b^	1.82 ± 0.45^b^	32.66 ± 7.53^b^	86.00 ± 21.50^b^	92.33 ± 17.83^a^	15.63 ± 2.37^a^	0.50 ± 0.12^a^	1.74 ± 0.15^b^
	3.6 µg/l	17.33 ± 2.60^a,b^	42.00 ± 7.63^b^	21.66 ± 2.60^b^	1.60 ± 0.11^a^	2.21 ± 0.57^b^	0.47 ± 0.04^b^	1.73 ± 0.55^b^	26.00 ± 4.35^b^	89.33 ± 13.44^b^	55.33 ± 0.88^b^	18.53 ± 2.16^a^	0.59 ± 0.07^a^	1.50 ± 0.11^b^

Values are represented as the mean ± SE. The means within the same column carrying different superscripts are significantly different

*ALT* alanine aminotransferase, *AST* aspartate transaminase

As shown in Table 4, fish exposed to both concentrations of PRO showed significant decreases in the serum levels of total protein, albumin, globulin, glucose, and total lipid on 7^th^ and 15^th^, and 21^st^ days. Serum cholesterol concentrations of fish showed significant changes after exposure to 1.8 and 3.6 µg/L of PRO on the 1^st^, 7^th^, 15^th^, and 21^st^ days. The serum urea and creatinine concentrations of *C. idella* showed significant increases after exposure to both concentrations of PRO over exposure periods (Table 4). On the 7^th^, 15^th^ and 21^st^ days, there were significant decreases in fish serum uric acid concentration after exposure to 1.8 and 3.6 µg/L of PRO.

As shown in Table 5, exposure of fish to 1.8 or 3.6 µg/L of PRO for 1, 7, 15 and 21 days induced significant decreases in the liver total protein and total lipids concentrations. However, on the 7^th^, 15^th^ and 21^st^ days, there was a significant decrease in the liver glycogen concentrations of fish exposed to both concentrations of PRO. Significant decreases were recorded in total protein, lipid concentration, and glycogen in muscles on the 7^th^, 15^th^ and 21^st^ day of exposure to both concentrations of PRO (Table 5).

**Table 5:** Hepatic and muscle protein, lipid, and glycogen concentrations as well as immunological parameters of *Ctenopharyngodon idella* exposed to different concentrations of PRO

Exposure time (day)	PRO concentration	Liver			Muscle			Serum	
		Total protein (g/100 g b.wt)	Total lipid (g/100 g b.wt)	Glycogen (g/100 g b.wt)	Total protein (g/100 g b.wt)	Total lipid (g/100 g b.wt)	Glycogen (g/100 g b.wt)	Lysozyme (µg/ml)	IgM (µg/ml)
1	None (control)	17.33 ± 2.02^a^	3.92 ± 0.61^a^	3.89 ± 0.11^a^	20.83 ± 2.38^a^	1.82 ± 0.16^a^	0.74 ± 0.05^a^	13.42 ± 0.62^a^	1.87 ± 0.22^a^
	1.8 µg/l	12.13 ± 1.11^c^	2.38 ± 0.27^b^	3.43 ± 0.12^a^	17.00 ± 2.30^b^	1.35 ± 0.20^a,b^	0.75 ± 0.08^a^	11.80 ± 0.30^b^	1.63 ± 0.06^a^
	3.6 µg/l	14.16 ± 1.74^b^	2.58 ± 0.38^b^	3.80 ± 0.79^a^	15.00 ± 2.30^b^	1.07 ± 0.18^b^	0.72 ± 0.04^a^	11.35 ± 0.55^b^	1.58 ± 0.04^a^
7	None (control)	17.22 ± 2.01^a^	3.88 ± 0.60^a^	3.92 ± 0.11^a^	21.24 ± 2.36^a^	1.88 ± 0.17^a^	0.73 ± 0.03^a^	13.36 ± 0.63^a^	1.86 ± 0.21^a^
	1.8 µg/l	12.83 ± 1.25^a,b^	2.23 ± 0.33^b^	3.53 ± 0.34^a,b^	13.33 ± 2.40^b^	0.93 ± 0.11^b^	0.66 ± 0.05^b^	10.75 ± 0.45^b^	1.51 ± 0.03^a^
	3.6 µg/l	10.27 ± 0.86^b^	1.58 ± 0.20^b^	2.60 ± 0.45^b^	11.33 ± 0.88^b^	0.85 ± 0.14^b^	0.58 ± 0.03^c^	10.80 ± 0.60^b^	1.49 ± 0.03^a^
15	None (control)	17.14 ± 2.03^a^	3.94 ± 0.64^a^	3.98 ± 0.12^a^	21.18 ± 2.40^a^	1.81 ± 0.17^a^	0.74 ± 0.06^a^	13.55 ± 0.65^a^	1.84 ± 0.22^a^
	1.8 µg/l	10.40 ± 1.47^b^	2.18 ± 0.40^b^	2.67 ± 0.37^b^	11.66 ± 2.18^b^	0.85 ± 0.14^b^	0.60 ± 0.04^a,b^	10.35 ± 0.45^b^	1.34 ± 0.01^b^
	3.6 µg/l	10.17 ± 1.47^b^	1.58 ± 0.33^b^	2.47 ± 0.37^b^	10.00 ± 1.15^b^	0.74 ± 0.05^b^	0.54 ± 0.03^b^	10.10 ± 0.60^b^	1.33 ± 0.01^b^
21	None (control)	16.83 ± 2.01^a^	3.93 ± 0.63^a^	3.84 ± 0.10^a^	21.46 ± 2.41^a^	1.78 ± 0.16^a^	0.75 ± 0.06^a^	13.26 ± 0.62^a^	1.88 ± 0.23^a^
	1.8 µg/l	8.26 ± 0.81^b^	1.51 ± 0.19^b^	1.70 ± 0.23^b^	9.00 ± 1.15^b^	0.61 ± 0.05^b^	0.51 ± 0.03^b^	9.40 ± 0.20^b^	1.29 ± 0.02^b^
	3.6 µg/l	8.27 ± 1.33^b^	1.23 ± 0.31^b^	1.66 ± 0.29^b^	8.67 ± 1.20^b^	0.56 ± 0.02^b^	0.46 ± 0.02^b^	9.20 ± 0.60^b^	1.27 ± 0.02^b^

Values are expressed as mean ± SD, *n* = 10. Values are represented as the mean ± SE. The means within the same column carrying different superscripts are significantly different

#### Immunological Studies

As shown in Table 5, on the 1^st^, 7^th^, 15^th^ and 21^st^ days of exposure to both concentrations of PRO, significant decreases were recorded in the serum total lysozyme concentrations of fish. However, on the 15^th^ and 21^st^ day, a significant decrease was recorded in the serum IgM in both concentrations of fish.

#### Histopathological alterations

**Fig. 1:**
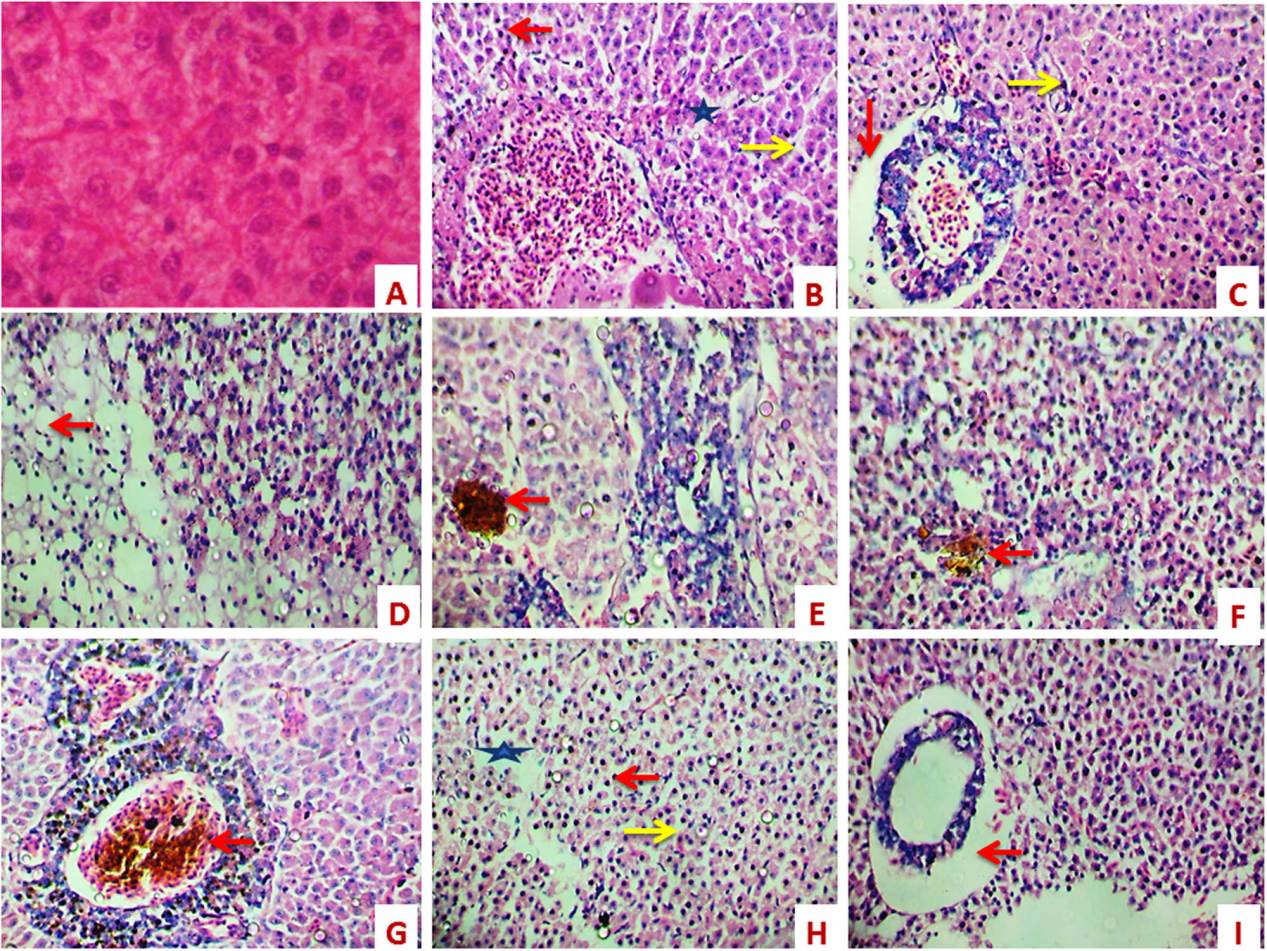
Sections of the liver of fish showing control (**A**) (X400), focal areas of necrosis (star) and coagulative necrosis of hepatocytes (yellow arrow) with nuclear pyknosis (red arrow) (**B**) (X400) (1^st^ day-1.8 µg/L of PRO), nuclear pyknosis (yellow arrow) and edema around the hepatoportal blood vessel (red arrow) (**C**) (X400) (1^st^ day-3.6 µg/ L of PRO), and vacuolar degeneration in the hepatocytes (red arrow) (**D**) (X400) (7^th^ day-1.8 µg/ L of PRO). Accumulation of hemosiderin between hepatocyte (red arrow) (**E**, **F**) (X400) 7^th^ day-3.6 µg/l and 15^th^ day-1.8 µg/ L of PRO. Dilation and thrombosis formation in the hepatoportal blood vessel (red arrow) (**G**) (X400) (15^th^ day-3.6 µg/ L of PRO). Focal areas of necrosis between hepatocytes (star), coagulative necrosis of hepatocytes (yellow arrow) with nuclear pyknosis (red arrow) (**H**) (X400) (21^st^ day-1.8 µg/ L of ORO) and edema around the hepatoportal blood vessel (red arrow) (**I**) (X400) (21^st^ day-3.6 µg L of PRO)

On day 1, after exposure to 1.8 and 3.6 µg/ L of PRO, the liver showed nuclear pyknosis. Moreover, moderate coagulative necrosis in the hepatocytes with a focal area of necrosis was observed (Fig. 1B). Also, edema around the hepatoportal blood vessels was noticed in fish exposed to 1.8 µg/L of PRO (Fig. 1C).

In fish exposed to both concentrations of PRO for 7 days, the liver showed vacuolar degeneration in the hepatocytes with focal areas of necrosis and nuclear pyknosis (Fig. 1D). Moreover, a slight accumulation of hemosiderin between the hepatocytes was observed in fish exposed to 3.6 µg/ L of PRO (Fig. 1E).

On day 15, after exposure to 1.8 and 3.6 µg/L of PRO, the liver showed moderate coagulative necrosis of hepatocytes. Moreover, slight accumulation of hemosiderin between the hepatocytes, focal areas of necrosis between the hepatocytes and vacuolar degeneration of hepatocytes were observed in fish exposed to 1.8 mg/ L of PRO (Fig. 1F). Also, dilation and thrombosis formation in hepatoportal blood vessels was noticed in fish exposed to 3.6 µg/L of PRO (Fig. 1G). Focal areas of necrosis between the hepatocytes and coagulative necrosis of hepatocytes were observed in the liver of *C. idella* exposed to both concentrations of PRO for 21 days (Fig. 1H). Moreover, edema around the hepatoportal blood vessels was noticed after exposure to 3.6 µg/ L of PRO (Fig. 1I).

**Fig. 2:**
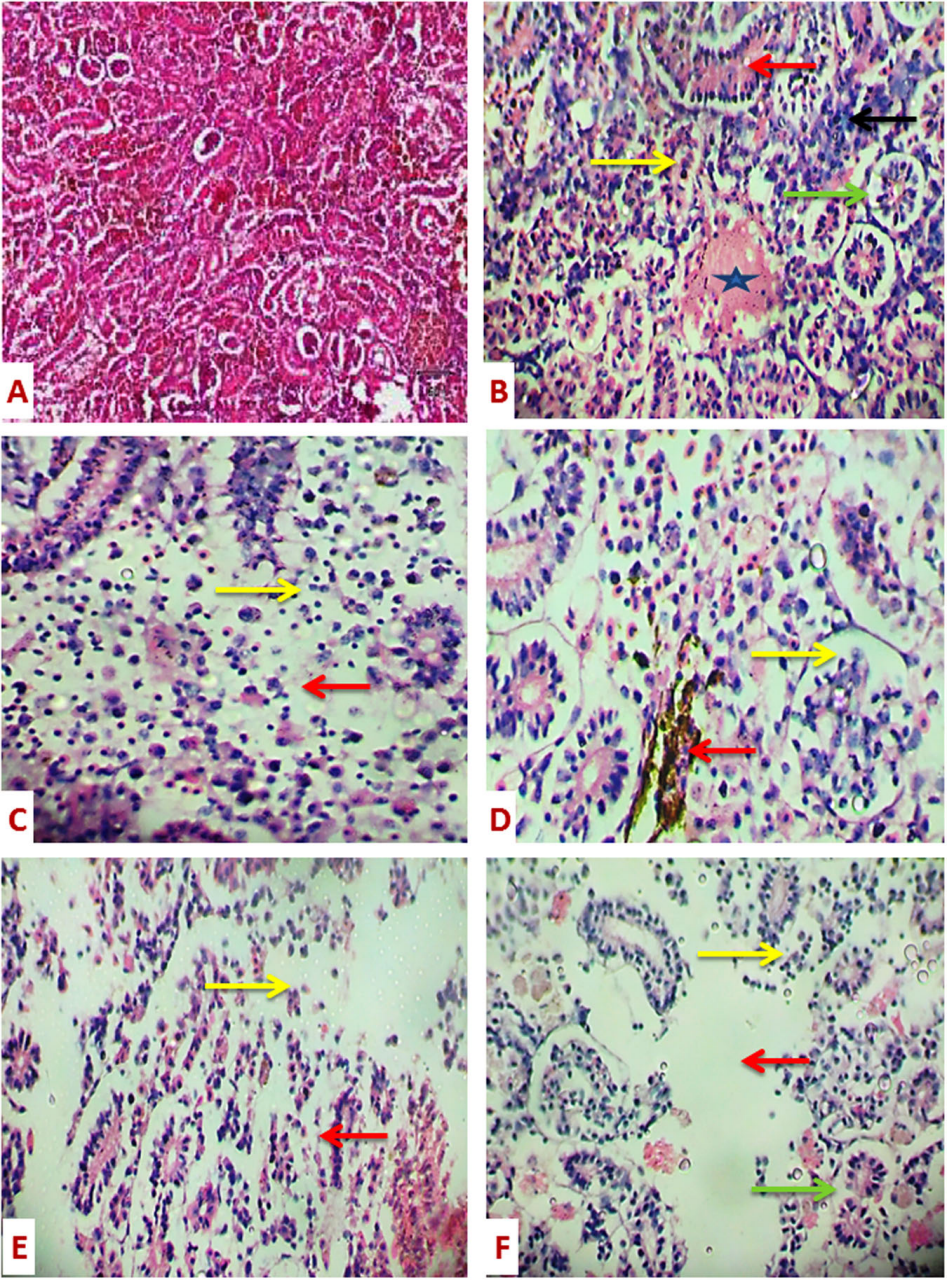
Sections of the kidney of *Ctenophyrogoden idella* stained with H&E showing control (**A**) (X100), narrowing of renal tubules (red arrow), aggregation of inflammatory cells between renal tubules (black arrow) and hemolysis between renal tubules (star) and vacuolar degeneration in the epithelial cells of renal tubules (green arrow) and degeneration of renal tubules (yellow arrow) (**B**) (X400) (1^st^ day −1.8 µg/L of PRO). Severe depletion of hemopoietic tissue (yellow arrow) and degenerative and necrotic changes in renal tubules with a focal area of necrosis (red arrow) (**C**) (X400) (1^st^ day −3.6 µg/L of PRO), accumulation of hemosiderin between renal tubules (red arrow) and edema in Bowman’s capsule (yellow arrow) (**D**) (X400) (7^th^ day 1.8 µg/L of PRO). Depletion of hemopoietic tissue (red arrow) and degenerative and necrotic changes with focal areas of necrosis (yellow arrow) (**E**) (X400) (7^th^ day −3.6 µg/ L of PRO). Vacuolar degeneration in the epithelium of renal tubules (green arrow), depletion of hemopoietic tissue (yellow arrow) and degenerative and necrotic changes with focal areas of necrosis (red arrow) (**F**) (X400) in both concentrations of PRO for 15 and 21 days

On day 1, after exposure to 1.8 and 3.6 µg/ L of PRO, the kidney showed degeneration of renal tubules. Moreover, narrowing of capillary tubes of renal tubules, hemolysis in the renal blood vessel and vacuolar degeneration in the epithelial cells of renal tubules with degeneration of renal tubules were observed in fish exposed to 1.8 µg/ L of PRO (Fig. 2B). Also, depletion of hemopoietic tissue was noticed after exposure to 3.6 µg/ L of PRO (Fig. 2C). Narrowing of capillary tubes of renal tubules, vacuolar degeneration in the epithelium of renal tubules and activation in the hemopoietic tissue, accumulation of hemosiderin between renal tubules and edema in Bowman’s capsule (Fig. 2D) were observed in fish exposed to 1.8 µg/ L of PRO for 7 days. Also, depletion in the hemopoietic tissue and degenerative and necrotic changes in the renal tubules with focal areas of necrosis were noticed after exposure to 3.6 µg/ L of PRO (Fig. 2E).

In fish exposed to both concentrations of PRO for 15 and 21 days, the kidney showed vacuolar degeneration in the epithelium of renal tubules, depletion in the hemopoietic tissue and degenerative and necrotic changes in the renal tubules with focal areas of necrosis (Fig. 2F).

**Fig. 3:**
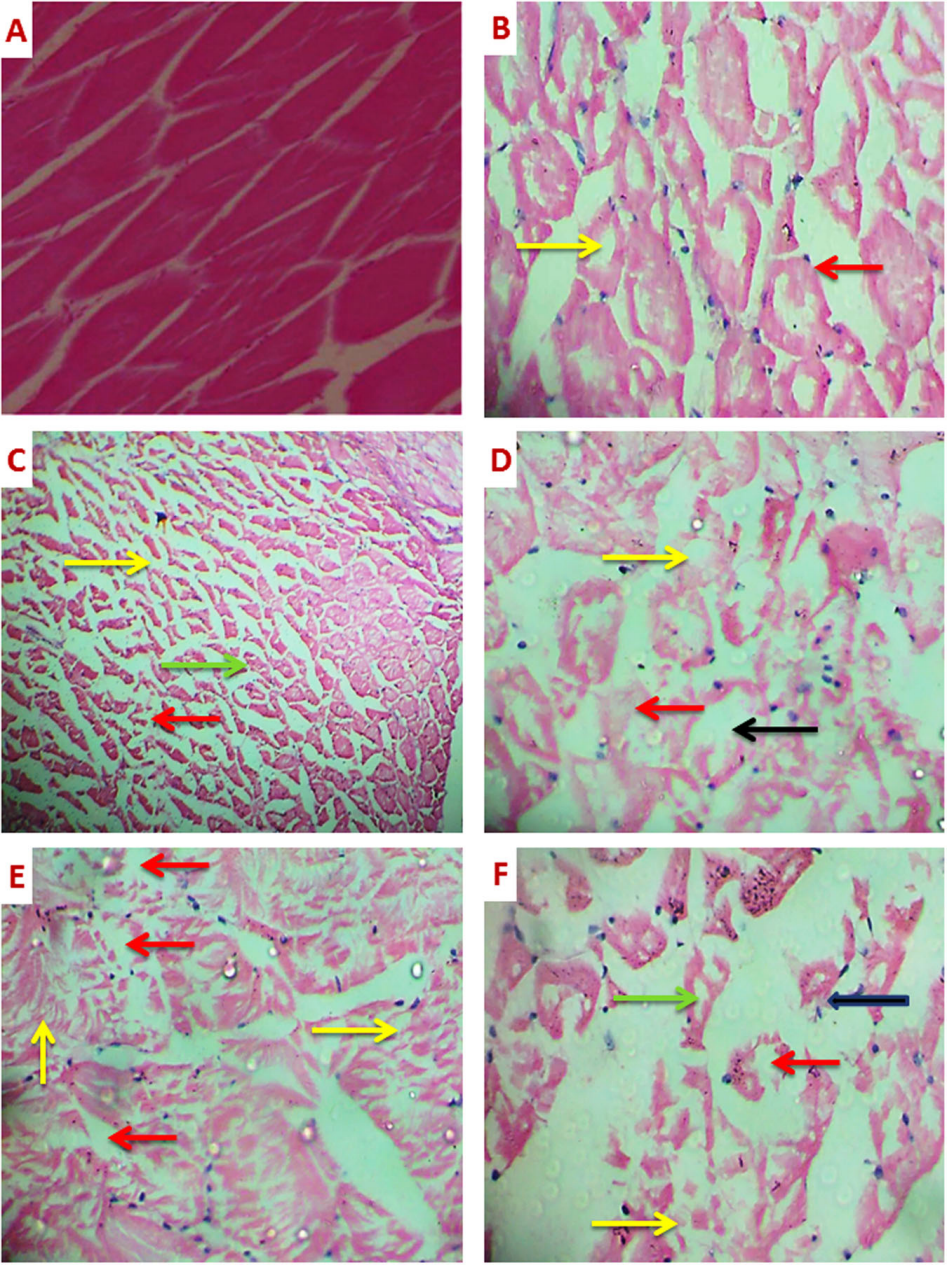
Sections of the muscle of *Ctenophyrogoden idella* stained with H&E showing control (**A**) (X100), vacuolar degeneration in muscle bundles (yellow arrow) and mild atrophy of muscle bundles (red arrow) (**B**) (X400) (1^st^ day 1.8 µg/L of PRO). Severe atrophy of muscle bundles (yellow arrow) and degeneration in muscle bundles with a focal area of necrosis (green and red arrows) (**C**) (X400) (1^st^ day −3.6 µg/L of PRO), severe degeneration in muscle bundles with a focal area of necrosis (yellow and red arrows) and vacuolar degeneration in muscle bundles (black arrow) (**D**) (X400) 7^th^ day of −1.8 µg/L and −3.6 µg/L of PRO and 15^th^ day −1.8 µg/Lof PRO). Splitting of muscle bundles (yellow arrows) and severe degeneration in muscle bundles with a focal area of necrosis (red arrows) (E) (X400) (15^th^ day −3.6 µg/L of PRO), severe atrophy of muscle bundles (black arrow), vacuolar degeneration in muscle bundles (green arrow) and degeneration in muscle bundles with a focal area of necrosis (red and yellow arrows) (**A**) (X400) 21^st^ day of 1.8 µg/l and −3.6 µg/l of PRO

On day 1, the muscles of fish exposed to 1.8 and 3.6 µg/ L of PRO showed mild atrophy (Fig. 3B) to severe atrophy of muscle bundles and vacuolar degeneration in muscle bundles (Fig. 3C). Moreover, degeneration of muscle bundles was noticed in fish exposed to 3.6 µg/ L of PRO. In fish exposed to both concentrations of PRO for 7 days, the muscle showed severe vacuolar degeneration in muscle bundles. Moreover, atrophy of muscle bundles. Also, focal areas of necrosis were observed (Fig. 3D). Exposure of fish to 1.8 µg/ L of PRO for 15 days induced vacuolar degeneration in muscle bundles. Splitting of muscle bundles and severe degeneration in muscle bundles with a focal area of necrosis were observed after exposure to 3.6 µg/ L of PRO (Fig. 3E). On day 21 of exposure to both concentrations of PRO, the muscle showed severe atrophy of muscle bundles, vacuolar degeneration in muscle bundles and degeneration in muscle bundles with a focal area of necrosis (Fig. 3F).

## Discussion

The application of the LC_50_ has gained acceptance among toxicologists and is the highest-rated test for assessing the potential adverse effects of chemical contaminants on aquatic life (Gad and Saad 2008; Khayatzadeh and Abbasi 2010). 96 h LC_50_ determination has been widely recommended as a preliminary step in toxicological studies on fish (APHA 2005; Parrot et al. 2006; Moreira et al. 2008). Fish mortality because of pesticide exposure mainly depends upon its concentration, sensitivity to the toxicants and exposure time duration (Kamble et al. 2011). The 96 h LC_50_ of PRO on grass carp was 0.0072 mg/L. The detection of LC_50_ concentration of pollutants is an important step before carrying out further studies on physiological changes in animals. The data on lethal toxicity provides useful information for identifying the mode of action of a substance and also helps in comparing dose-response among different chemical substances. The acute toxicity of organophosphate (PRO) was studied on many species like *Oreochromis mossambicus* (Nair 2006), *Cyprinus carpio* (Joseph and Raj 2011) and *Oreochromis niloticus* (Sharafeldin et al. 2015). To our knowledge, this is the first study on grass carp.

Herein *C. idella* exposed to PRO swam erratically and very rapidly with a semi-circular swimming behavior and tried to jump out of the aquarium, increased opercular movement, aggressive behavior, rapid gulping of water, knocking the wall of the aquarium. Similarly, Rahman et al. (2002) observed nearly the same clinical signs. This was attributed to respiratory impairment and irritation due to toxicant in water which affects gills. Additionally, the observed difficulty in respiration mainly reflects a decreased respiratory capability because of the damage to the gills. In later stages of exposure, the exposed excited fish laid on their sides on the bottom of the aquarium, making very slight movement and remaining motionless on the aquarium bottom until death.

In the present investigation, *C. idella* showed considerable alteration in different blood parameters after exposure to sublethal concentrations of 1.8 and 3.6 µg/L of PRO. A significant decrease in Hb percentage, total erythrocyte count, and hematocrit values indicate the occurrence of anemia associated with erythropenia. The anemia may be due to the inhibition of erythropoiesis and hemosynthesis and to an increase in the rate of erythrocyte destruction in hemopoietic organs such as the kidney, as proved by histopathological studies. Reduction in Hb level may be the consequence of the toxic effects of PRO on the synthesis of this molecule. The toxicant may inhibit the synthetic pathway by affecting the activity of enzymes involved in the Hb synthesis. Similar findings were recorded in *Labeo rohita* exposed to lethal and sublethal concentrations of PRO (Zenebehagos et al. 2017) and in Nile tilapia, *Oreochromis niloticus* (Khan 2019). Erythrocytes decrease has been reported by Abdelmeguid et al. (2002) in Tilapia Zilli due to water pollution. The damage caused to the intestine by the toxicant may be a reason for impaired iron absorption that led to its deficiency, as reported by (Joshi et al. 2002). Erythrocytes are crucial for determining fish exposed to toxins’ structural and functional status. Erythrocytes can react to a few environmental stresses, and changes in fish erythrocytes (both nuclear and cellular) are the most frequent sign that pesticides are present in a body of water (Sawhney and Johal 2000). Various erythrocytic abnormalities such as binucleated, tear-drop-shaped, microcytic, and hypochromic erythrocytes, were revealed, indicating PRO toxicity. Various erythrocytic abnormalities such as binucleated, elongated shaped, tear-drop shaped, and twin were found in stained blood smears of Tilapia treated with different PRO concentrations (Khan 2019). The decline in PCV might be due to shrinking cell size after intoxication. The decrease in MCV was reasoned for the variation in red cell volume attributed to exosmosis indicated by increased electrolyte concentration inside the red cell after insecticide treatment (Reddy et al. 1991). Because MCH and MCHC are derived from Hb and RBC, any alteration in Hb and RBC levels would lead to the alteration of MCH and MCHC. It was reported that MCV indicates the status or size of RBCs (Alwan et al. 2009). We noticed a significant decrease in MCV, MCH, and MCHC concentrations in *C. idella* treated with PRO. A significant decline in MCH and MCV levels indicates hypochromic microcytic anemia. Similar anemia was produced due to PRO intoxication in *Labeo rohita* (Zenebehagos et al. 2017).

In the present investigation, WBC count concentration increased on all days of exposure periods to PRO. An increase in the WBC count may result from direct stimulation of immunological defense due to a toxic substance or may be associated with induced tissue damage. The increase of leucocytes (WBC) count of treated fish reflects a general state of toxemia exhibiting impairment of the defense mechanism and is manifested into leukocytosis to cope with such a situation. Similar results were reported in teleosts by Ramesh and Saravanan (2008) on exposure to different pesticides. Enhanced WBC count in *L. rohita* has been reported when exposed to profenofos (Kesharwani et al. 2017). Moreover, Shrafeldin et al. (2015) and Al-Emran et al. (2022) revealed a significant increase in WBCs counts during both acute and chronic exposure to profenofos on Nile tilapia.

Serum total proteins were useful in diagnosing fish disease (El-Houseiny et al. 2022b). Most serum proteins are impaired by nitrogen metabolism (Murray et al. 1990). It is an indicator of liver impairment (Yang and Chen 2003). Elevation in serum total protein is possibly due to several pathological conditions such as damage to the liver and kidney, relative changes in the mobilization of blood proteins, activation of metabolic systems in response to pesticides exposure, degradation of the cellular material in the liver, water loss in the serum and induction of protein synthesis in the liver. Later, significant decreases were recorded in the serum total protein, albumin and globulin concentrations after exposure to PRO. The reduction in protein, albumin and globulin concentrations of the serum in this study may be because the liver function may be impaired and no longer produce albumin or proteins. The total protein was reported as an index of liver disturbance by (Yang and Chen 2003). Reduction in the total protein level of serum due to PRO toxicity has been reported by Nagaraju and Rathnamma (2013). Firat et al. (2011) attributed the reduction in total protein to the damaging effects of pesticides on liver cells. Herein, the decrease in glucose level might be due to the increase of glucose oxidation to meet the higher energy demands during chronic exposure. Similar findings have been recorded by Al-Emran et al. (2022) on Tilapia fish after exposure to PRO. Since carbohydrates serve as the instant energy source during stress, during the acute condition, blood glucose level increases due to glycogenolysis, but reduction can be correlated to the utilization of stored glycogen to meet the energy demand or chronic exposure. In the liver, glycogen mobilized to glucose, whereas in muscle, glycogen/glucose served as readily available energy; thus, hypoglycemia was observed. The increase of cholesterol observed in the present study may be due to one or more of the following reasons: increased production by the liver and other tissues by the effect of the pesticides, release of cholesterol from damaged cell membranes, decreased hepatic excretion of cholesterol, thyroid dysfunction and finally blocked conversion of cholesterol to sex steroids as a result of gonad dysfunction and decreased activity of cytochrome P450 enzymes (Metwally 2009). The serum cholesterol level has been observed to be decreased after PRO treatment for different time intervals (Sharafeldin et al. 2015). The decline in cholesterol levels after that is due to the utilization of stored and circulatory cholesterol and other lipid fractions in pesticide-treated fish to counteract the toxic effect produced and further stabilize the toxic pesticides to prevent harm caused by them. This is mainly due to altered lipid metabolism and energy demand. The reduction in total lipid was explained as a direct effect of the utilization of body fat as an energy supply to meet the increasing physiological demands and as a result of pollutant stress which enhanced metabolic rate and reduced metabolic reserves. This may be supported by the current depression of total protein and glycogen content in muscle and liver (Sharafeldin et al. 2015).

In the present study, serum urea and creatinine significantly increased in *C. idella* after exposure. This result supported that PRO exerts harmful effects on the kidney tissues causing kidney dysfunction. It is well known that renal insufficiency or failure is usually associated with decreases in urea, uric acid, and creatinine excretion, thus leading to increases in serum. The elevation of urea level may be attributed to gill dysfunction **(**Stoskoph 1993). Chang et al. (1996) concluded that kidney damage might result in reduced renal blood flow with a reduced glomerular filtration rate, resulting in azotemia characterized by increased blood urea and creatinine. Urea in fish is synthesized by the liver and excreted primarily by the gills rather than the kidney. It is shown that the increased blood urea could occur at times of impaired kidney function, liver diseases and cardiac arrest (Abdelmoneim et al. 2008). The histopathological alterations in the studied fish’s liver, kidney, and gills supported the increased serum urea and creatinine level. In contrast, there was a decrease in the uric acid of fish after exposure to both concentrations of PRO on all days of exposure. Low uric acid levels have no significance, but their increase indicates several disturbances in the kidney (Maxine and Benjamin 1985).

The serum ALT and AST concentrations decreased in *C. idella* after exposure to both concentrations of PRO. On the other hand, ALP decreased after exposure to both concentrations of PRO except on 1^st^ day after exposure. Reductions in ALT, AST and ALP values in fish exposed to various toxicants have been reported previously (Banaee et al. 2008; Gabriel et al. 2012). Impairment of the serum membrane of the liver may be the reason for the reduction in ALP activity in the liver and, subsequently, the sera of the tested fish. The lower values of AST, ALT and ALP enzyme activities were suggested by inactive transamination and oxidative deamination (Gabriel et al. 2012) and the inhibition of intermediary metabolic processes (Begum 2005). Adeyemi et al. (2008) also believed that any alteration at the subcellular level might affect the activity of ALT and AST enzymes. The activity of enzymes produced in the liver is consistent with the serum protein concentrations in this experiment.

The obtained results showed an appreciable decline in different biochemical constituents of the fish tissues (glycogen, total lipid and total protein levels in liver and muscle) under pesticide stress. In the present study, the results clearly indicated a decrease in glycogen content to resist the effects of pesticides. The decrease in the glycogen concentration of the tissues of *C. idella* can be due to its enhanced utilization as an immediate source to meet energy demands under PRO stress. The glycogen content was observed in a decreasing manner with increasing concentrations. Because of the stress, the fish make suitable adjustments for which the stored energy is utilized. This may be the reason for the decreased amount of glycogen content consumed to provide immediate energy to the body’s fighting elements and protect all body systems from the harmful effect of pesticides. It could also be due to the prevalence of hypoxic or anoxic conditions, which normally enhances glycogen utilization **(**Dezwaan and Zandee 1973). Depleted glycogen levels following chromium stress were reported in *Cyprinus carpio communis* (Ambrose et al. 1994) under hypoxic conditions and in *O. niloticus* exposed to PRO (Shrafeldin et al. 2015). The protein content in the muscle and liver of *C. idella* is decreased with increasing concentrations of PRO. The decrease in protein content indicates that the tissue protein undergoes proteolysis. The decrease in tissue lipids and proteins might be partly due to their utilization in cell repair and tissue organization with the formation of lipoproteins, which are important cellular constituents of cell membranes, and cell organelles present in the cytoplasm (Harper 1983). A decrease in the lipid concentration observed in the present study can also be attributed to its utilization in cell repair and tissue organization. Moreover, the decrease in the total lipid of the liver with the increase in the concentration of PRO may be attributed to the utilization of energy storage to meet more energy demands for the detoxification process and also to balance the hindrance of normal metabolism (Bawa et al. 2017). Similar effects of PRO on total protein and lipids in Tilapia were demonstrated by Shrafeldin et al. (2015).

In our study, there was a significant decrease in lysozyme activity in serum of fish exposed to PRO. This result is in accordance with the study of Rahman et al. (2020), who observed a significant decrease in lysozyme activity in the plasma of *Cyprinus carpio* exposed to PRO. There were no significant changes in serum IgM of fish exposed to PRO all over exposure periods except in fish exposed to 3.6 µg/L of PRO on the 21^st^ day. Similarly, Girón-Pérez et al. (2008) found that the IgM levels in plasma from Nile tilapia exposed to 0.39 and 0.78 mg/L diazinon were not affected. But at 1.96 mg/L, diazinon increased IgM concentrations. This finding agrees with a previous study in which intermediate doses were tested (Garg et al. 2004).

Tissue histology is regarded as a marker of exposure to pollutants and is an effective tool to evaluate the pollution level, particularly for sublethal impacts (El-Houseiny et al. 2022c). In the present study, histopathological data revealed that profenofos exhibited tissue alterations in fish liver, kidney, and muscle. Several changes were produced, such as coagulative necrosis of hepatocytes with nuclear pyknosis, vacuolar degeneration, and accumulation of hemosiderin between hepatocytes. Changes in the liver could be because the liver is the main site of detoxification, and it is expected that the toxicant would reach there abundantly for detoxification and disposal (Mushigeri and David 2005). The inability of fish to regenerate new liver cells may also have led to necrosis of hepatic cells of sinusoids. The renal tissue showed narrowing of renal tubules, aggregation of inflammatory cells and hemolysis between renal tubules, degenerative and necrotic changes, severe depletion of hemopoietic tissue, and accumulation of hemosiderin between renal tubules. Lesions recorded in the kidney indicate nephrotoxicity caused by the tested compound and its metabolites since kidneys are the way to eliminate most of the organophosphorus compound. Vacuolar degeneration, severe atrophy and degeneration in muscle bundles with a focal area of necrosis were observed in muscles. Our results parallel findings on the histological changes of different fish species exposed to pesticides **(**Mohamed et al. 2019). Various liver and kidney alterations were revealed in PRO-exposed *Cyprinus carpio* (Rahman et al. 2020) and in PRO-exposed *Egretta alba* (Taha 2022).

## Conclusion

Along these lines, PRO in the aquatic medium is a major factor responsible for drastic changes in the fish blood and tissues. Abnormal behavior, hypochromic microcytic anemia, leukocytosis, and negative biochemical and histopathological effects on the liver, muscles, and kidney were common features of fish health status due to PRO toxicity. So, this issue must be considered during toxicological analysis and control of the main aquatic pollutant.

## Supplementary information

Supplementary Figure

Supplementary Figure Legend

## Data Availability

Data of the present article are available under request.
