# Supplementary figures and images for: Toxicity bioassay and sub-lethal effects of profenofos-based insecticide on behavior, biochemical, hematological, and histopathological responses in Grass carp (Ctenopharyngodon idella)
Source: Ecotoxicology. 2023 Jan 28;32(2):196–210. doi: 10.1007/s10646-023-02628-9 (PMC10008772; doi:10.1007/s10646-023-02628-9)

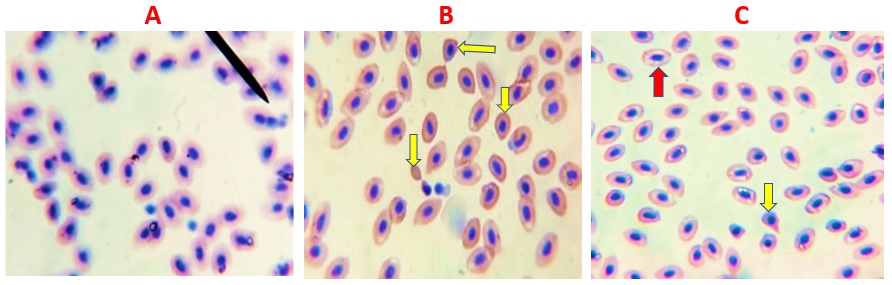

Supplement: Supplementary file 1 — Supplementary Figure [file 10646_2023_2628_MOESM1_ESM.jpg]
