# Supplementary material for: Toxicity bioassay and sub-lethal effects of profenofos-based insecticide on behavior, biochemical, hematological, and histopathological responses in Grass carp (Ctenopharyngodon idella)
Source: Ecotoxicology. 2023 Jan 28;32(2):196–210. doi: 10.1007/s10646-023-02628-9 (PMC10008772; doi:10.1007/s10646-023-02628-9)

Stained blood smear showed different abnormalities in blood cells of *Ctenopharyngodon idella* after exposure to PRO (A) binucleated cell (B) micronuclei and microcytic cells (C) Tear like cells (yellow arrow), hypochromic cells (red arrow).


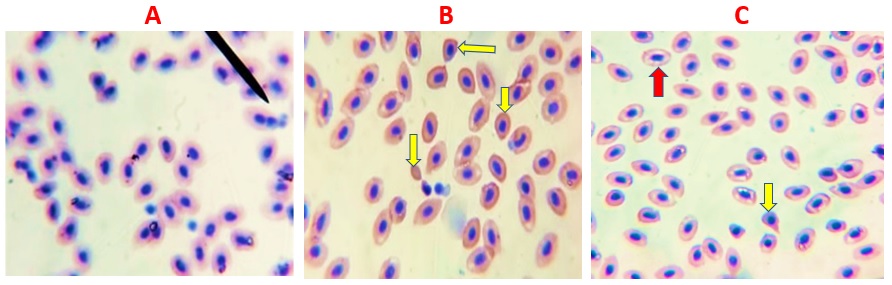

Supplement: Supplementary file 2 — Supplementary Figure Legend [file 10646_2023_2628_MOESM2_ESM.docx]
